# On Disorders of the Cerebral Circulation, and on the Connection between Affections of the Brain and Diseases of the Heart

**Published:** 1846-07

**Authors:** 


### On Disorders of the Cerebral Circulation, and on the Connection between Affections of the Brain and Diseases of the Heart. By *George Burrows*, M.D. 8vo. pp. 280—Plates. London, 1846.

The substance of this work has already been made known to a portion of the profession in the Lumleian Lectures delivered by the author in 1843 and 1844; but the subject treated of is one of such high importance, and the conclusions arrived at must, if admitted as sound, enforce so great a modification in the management of a large class of dangerous diseases, that an extended publication and criticism of the doctrines advanced is called for.

The emancipation of the mind from the despotic influence of great names is always a difficult and sometimes a very delicate task. There prevails so natural a feeling of veneration and gratitude for those who have preceded us in the paths of laborious investigation, and so many of their dogmas and principles are supported with able reasoning, well devised experiment, or sagacious observation, and have firmly resisted attacks from all quarters, that when any young practitioner (and it is ordinarily such who undertake opposition to any prevalent opinion) ventures to suspect that some of these may be fallacious, he is apt to be met with sneers or rebukes, and although he may have reason and truth on his side he will oftentimes fail in securing their reception. The difficulties, too, in breaking up a routine practice, however erroneously this may be based, can only be appreciated by those who have attentively observed the tenacity with which old prejudices and practices adhere even to well-informed persons, to say nothing of that preponderating class in all communities who continue to mechanically perform certain actions because they always have done so. When, however, we look back upon the history of medicine, and find that hardly a doctrine or theory once advocated by the ablest observers, holds its ground, and that what were once considered by acute men as axioms in practice, are now discarded as sheer fallacies by the merest tyro, we see the necessity of ever and anon re-examining the foundations of our medical creed, and submitting it to the reforms and improvements which the progress of science, the increased means of investigation, and the accumulation of facts have placed at our disposal. Still, it required some courage on the part of Dr. Burrows to announce to the assembled sages and seniors of his college that the principles upon which their practice in an important class of maladies is founded are erroneous, and to inculcate the absolute necessity of adopting measures of investigation to the use of which several of them are incompetent: but in his lectures there is an absence of self-sufficiency, a deferential mention even of the authorities he feels called upon to differ with, and an earnestness of tone in what he advances, that preserve him from the imputation of being a mere innovator, and enforce his claims to attention. Disciplined under the auspices of one of the best clinical teachers this metropolis has ever possessed, and furnished with the wide field of observation a large hospital alone can give, he has advanced no opinion without mature consideration and examination, and has advised no departure from routine practice without furnishing ample evidence of its inefficiency or its hurtfulness.

The work is divided into two portions. In the first of these, the erroneous doctrines respecting the cerebral circulation which have been commonly entertained are examined and refuted. In the second, the frequent dependance of functional and structural cerebral affections upon a diseased condition of the heart or its membrane is set forth.

I. *On the Circulation of the Brain.*—The opinion that *the brain under all circumstances of health and disease contains absolutely the same quantity of blood*, seems so contrary to common sense and daily experience, that its disproval would seem scarcely to require the careful elaboration Dr. Burrows has bestowed upon it; but when we consider it has been and is entertained by men of the highest eminence in the profession, when names such as Monro, Abercrombie, and Clutterbuck can be cited in its favour, it is evident that something beyond a mere negation of its correctness is called for. Its defence is based upon the mechanical structure of the cranium, and experiments upon animals. The cranium, it has been said, is a perfect sphere, the contents of which are protected from the effects of atmospheric pressure. Allowing that the bony case prevents that direct effect of pressure upon its soft contents which some other portions of the body are exposed to, the indirect effect exerted upon the blood entering at the orifices of the base is no less obvious.

In regard to the experiments upon animals, those which were performed by Dr. Kellie, and recorded in the first volume of the Medico-Chir. Trans. of Edin., have been quoted, first by Abercrombie, and subsequently by various other writers. The following are Dr. Kellie's propositions.

"1. That a state of bloodlessness is not discovered in the brains of animals which have died by hæmorrhage; but, on the contrary, very commonly a state of venous cerebral congestion. 2. That the quantity of blood in the cerebral vessels is not affected by gravitation, or posture of the head. 3. That congestion of the cerebral vessels is not found in those instances where it might be most expected; as in persons who die by hanging, strangulation, suffocation, &c. 4. That if there be repletion or depletion of one set of vessels (arteries or veins) in the cranium, there will be an opposite condition of the other set of vessels."

Dr. Burrows submits these experiments of Dr. Kellie to a renewed examination, and is naturally surprised at finding that, in contradiction to the *first proposition*, instances of a sheep and dog are given, whose brains, after they were bled to death, contained considerably less quantity of blood than did that of a dog who was killed by tying the jugulars and carotids. In the face of such a contradiction Dr. Burrows saw no other resource than the institution of new experiments. He killed two rabbits, the one by opening the blood-vessels of the throat, and the other by strangulation. On examining the brains of each, 24 hours after, he found that of the former completely blanched, and that of the other as completely congested. Plates representing these conditions are appended to the volume. "In fairness to Dr. Kellie I should state, that I have attended at the slaughtering of sheep by butchers, and find the brains of those animals much less depleted than the brains of rabbits which have died of hæmorrhage. But the sheep did not die of simple loss of blood; but partly from the division of the pneumo-gastric nerves and cervical portion of the cord. These lesions, no doubt, influenced the appearances." The experiments which Dr. Burrows has made in reference to the influence of *posture* are also in contradiction with the conclusions of Dr. Kellie; for animals placed under the same circumstances after death, excepting the dependent condition of the head, presented very different appearances of the brain as regards congestion.

"It may now be asserted that the encephalon is not exempted from the law in physics—the gravitation of the fluids to the lowest part of the corpse. The discovery of the operation of this force on the blood within the cranium after death, suggests a precaution very essential to be followed, when it is desired to ascertain the precise amount of congestion of the cerebral vessels at the time of death. In such cases, a ligature should be placed around the throat, and drawn sufficiently tight to compress the cervical vessels, and arrest all flow of blood through them. This precaution will be most required in the examination of bodies, where, from the kind of death, the blood may be suspected to remain fluid in the heart and great vessels. The depending or elevated position of the head during the examination of the body, will not then induce deceptive appearances, which mislead us in our conclusions as to the previous amount of congestion in the cerebral vessels." P. 21.

To support this third proposition, Dr. Kellie adduces some instances of the brains of persons who had been hung presenting no remarkable congestion. Dr. Burrows, although maintaining that congestion of the cerebral vessels is generally produced by this or any other description of death which kills by obstructing respiration, admits that such congestion is occasionally absent. He attributes this to the rope having imperfectly compressed the external jugular on one side, and still more to the subsidence of the fluid blood during the subsequent suspension of the body, through the unobstructed cervical vessels, as also through the vertebral sinuses and spinal veins. In examining the bodies too, of those who have died from strangulation, the neck is usually cut across, and the thoracic organs inspected prior to the brain, the blood from which last thus becomes discharged through the divided cervical vessels.

Dr. Kellie's fourth proposition is founded upon the assumption that, the amount of blood contained within the cranium is always the same. This, we have seen, Dr. Burrows denies; and therefore does not admit the necessity of the adjustment of the equilibrium between the two classes of vessels. He believes, indeed, that the amount of *extra-vascular serum* in the cranium is very variable, depending upon the amount of blood in the arteries or veins being more or less than normal.

*On Vascular Pressure within the Cranium, and its Influence on the Functions of the Brain.*—Dr. Abercrombie denied the existence of such pressure—first because " the cerebral substance is composed of inelastic fluids which are incompressible ;" and 2, " because the brain is incompressible by any such force as can be conveyed to it from the heart through the carotid and vertebral arteries." In reply to the first of these, Dr. Burrows observes that, the brain, although very incompressible is highly elastic. The existence of an outward pressure produced by the passage of the blood into the vessels is seen when a portion of the cranium has been destroyed by accident or disease. Movements of the brain, dependent upon the arterial pulse and upon the respiration, have long been observed and commented upon under these circumstances. Whatever diminishes the accession of arterial blood to the brain, diminishes also the former of these, while ligatures around the external jugulars much enfeeble the latter. In the ordinary condition of the cranium this pressure is reflected back upon the brain.

" In conclusion, I believe it to be most important to bear in mind, when considering various pathological states of the brain, that its substance not only contains a variable amount of blood at different times, but that it is also subjected to a constant vascular pressure. This pressure arises partly from arterial and partly from venous distension : also, it is increased during expiration, and diminished during inspiration : and although the substance of the brain is very unyielding and incompressible, nevertheless it sustains, and is influenced by, this vascular pressure. I consider it essential, in studying the physiology or pathology of the brain, to have constantly in remembrance the existence of this vital force. Now vital forces, just as the most efficacious remedies, when they exceed or fall short of their proper amount, are capable of producing the most serious ill effects in the animal economy. Numerous causes may affect this momentum of the blood in the vessels of the head, and hence give rise to very different degrees of vascular pressure : at one time it may become excessive, oppress the organ, and suspend its functions : at another time, it is insufficient, and seems to be inadequate to sustain the cerebral functions. The injurious effects of modifications of this pressure on the brain would be much more often exhibited, were it not for the ample development of the venous system in the cranium and spinal canal, which affords such ready exit for redundant blood ; and for another peculiarity in the anatomy of the parts contained in the cranium. The peculiarity to which I now advert, is rarely pointed out by teachers, and is not sufficiently estimated by pathologists. I allude *to the large amount of extra-vascular fluid in the cranium*, even in health, and which, in the form of serum, is found in the ventricles and membranes of the brain, as well as disseminated through its substance. This fluid, very appropriately designated *cephalo-rachidean*, or *cerebro-spinal*, varies greatly in amount at different times : and, from the anatomy of the parts, as well as from experiments, it would appear that a portion of this fluid readily changes its site from the cranium to the spinal canal, and conversely." P. 50.

The existence of this serum during life has been shewn by the experiments of Magendie, Longet and others, and anatomy exhibits the free communications which exist between the ventricles and the cavities of the spinal and cerebral membranes. The vivisections of Ecker and others shew us also that the situation of this fluid may be subjected to considerable change by modifications of pressure. Dr. Burrows regards this fluid as supplemental to the other contents of the cranium—increasing or diminishing in quantity inversely to the quantity of blood and serous matter contained within it. It may also be a means of equalizing pressure over the whole cerebro-spinal mass. When arterial or venous congestion of the brain is suddenly induced, the increased pressure which is exerted expels a portion of this serum into the spinal canal, while, when blood is abstracted from the cranium, the vacated cranial space is occupied by the spinal serum. When the cranium is healthy and normal, the cerebral substance may in this way accommodate itself to, a temporary increase of blood ; but when the increased determination to, or obstruction of return of blood from, the brain becomes more permanent, or the cranium contains other abnormal substances, the additional pressure will not be borne. Whenenever there is an increased amount of solid matter in the cranium, whether from hypertrophy, tumours, extravasation, &c., whatever excites the heart's action and temporarily increases the degree of vascular pressure must increase the disturbance of the functions of the brain. "It seems to me probable that many permanent structural lesions within the cranium do not affect the functions of the brain by pressure, except when there is some cause in operation capable of inducing vascular congestion, or when the lesion is of a mechanical nature, or is gradually increasing."

On the other hand, whatever diminishes the heart's power in these morbid conditions of the brain mitigates the cerebral symptoms : but if such deficiency prevails in the healthy state of the brain, this organ suffers then from *insufficient vascular pressure*, and *syncope* results, this state being produced by such deficient pressure of the brain, and not, as usually supposed, from the inadequate supply of blood furnished to it. Thus, in its most simple form, it may be induced by a mere moral emotion diminishing the heart's energy, so that the blood is not propelled with sufficient energy to maintain an adequate pressure upon the cerebral substance. On account of the additional labour imposed upon the heart, the syncope is more likely to occur if the person is erect, and is relieved by his assuming the horizontal posture. The greater rapidity with which this state is induced by the abstraction of blood in the erect posture proves that the posture rather than the amount of blood lost is the efficient cause.

"I am the more anxious to direct attention to the foregoing explanation of the phenomena of syncope, because a very different opinion has been advanced in a recent work (Lib. of Medicine, Vol. 2) in extensive circulation among the junior members of the profession. Thus, in an Essay on Apoplexy (p. 92), it is asserted that syncope differs from apoplexy only in the extreme feebleness of the heart's action ; but the cause producing loss of consciousness, sensation, and motion, is stated to be the same in both affections. In either case, it is said, owing to the peculiarities of the circulation within the cranium, *pressure* is exerted on the brain ; and in some cases it is difficult to distinguish the states of apoplexy and syncope from each other. 'Thus either from increased or diminished action of the heart, pressure on the brain may be produced by over-distension of its vessels ; in the first case, of its arteries, and in the second, of its veins.' Here we find it promulgated that apoplexy and syncope are to be attributed to the same physical cause, viz. pressure on the brain ; and that in syncope the pressure arises from the diminished action of the heart occasioning fulness of the cerebral veins. Now, I believe, that so far from syncope being occasioned by pressure on the brain, it will be found, as I have stated at some length, that every method of diminishing vascular pressure on the brain to any great extent, whether it be accomplished by depressing moral emotions, by sudden loss of blood, by the erect posture of the body, or by contrivances which diminish the momentum of the blood flowing towards the brain, will almost certainly induce syncope.

"But if syncope be produced by venous congestion, causing pressure on the brain, would any practitioner of experience attempt to overcome it by the use of those remedies most likely to diminish venous congestion of the brain, and the consequent pressure on that organ? Would he be bold enough to place his fainting patient in the erect posture, or draw blood from the jugular vein? I have also shown by experiments, that when an animal is bled to the point of fatal syncope, that, so far from finding venous congestion of the brain after death, all its vessels, are, on the contrary, ex-sanguine. It appears to me, that syncope differs from apoplexy in every respect but in this one, viz. that in both there is a total temporary abolition of the functions of the brain. The causes producing the abolition, and the means to be employed to restore the functions of the brain, are generally quite opposite. Without presuming to be hypercritical, there is cause to regret that such erroneous doctrines as to the nature of so alarming a condition of the system as syncope should have been disseminated by modern writers." P. 64.

Dr. Burrows does not attribute the disturbance of the functions of the brain which takes place in *anæmia*, so much to the actual deficiency of blood in the organ, as to the defective amount of vascular pressure exerted upon it. Thus, in *anæmia from hypertrophy of the brain*, in which the dense cerebral mass is dry and destitute of blood, none of these symptoms are present: but in general anæmia, resulting from loss of blood, the various severe nervous symptoms are all at least temporarily mitigated by posture, stimuli, and whatever favours the momentum of the blood entering the brain.

The effects which have resulted from the *ligature of one or both carotids* also illustrate the doctrine here laid down. Mr. Key tied the right common carotid in a woman æt. 61, and she died in four hours, having manifested stertorous breathing. The carotid on the right side was found nearly obstructed and the vertebrals small. The brain was healthy, and the sudden diminution of the momentum of the blood in the cerebral arteries seems the only mode of explaining her rapid death. Although such a result does not usually follow the ligature of one common carotid, yet a reference to the numerous cases, collected by M. Longet and Dr. Chevers, show the frequency of the speedy supervention of marked disturbance of the cerebral functions or the subsequent occurrence of hemiplegia dependent upon cerebral disorganization.

"These subsequent phenomena appear to me to arise from two causes; partly from the insufficient supply of blood to the disorganized hemisphere of the cerebrum, and partly from the compression of the exsanguined hemisphere by its fellow, the vessels of which still continue to be liberally supplied with blood. In healthy states of the circulation within the cranium the forces distending the blood-vessels in either cerebral hemisphere are equal, opposite, and counterbalance each other: but so soon as the free supply of blood to one hemisphere is cut off by the ligature of the common carotid, the vascular distension in the other hemisphere becomes a source of pressure on the exsanguined side.

Hence probably the cause of the commencing hemiplegia, which gradually increases with the disorganization of the cerebral substance. I am inclined to attribute the successful termination to this operation in some cases to the opportune loss of blood from a wound in the throat prior to the application of the ligature;* or the same happy result may be ascribed to a cautious preparatory venesection before the common carotid has been secured. By such loss of blood the circulation has been quieted, and the difference in the momentum of the blood in different parts of the brain has been, if not obviated, at any rate very much diminished. It might be objected that the effects upon tying one of the carotids in the human subject are too diversified to admit of the foregoing explanation; but, in reply to such objection, it may be urged, that, although certain effects are usually produced by large abstractions of blood, still the results of blood-letting are very dissimilar in different individuals." P. 77.

The effects which result from obstructing the circulation of blood through the carotids have induced some practitioners to seek from it a remedial agency. Thus, Mr. Prescott of Calcutta, has tied the carotids for the relief of severe *epilepsy* and *cephalea* in two cases; and Dr. Parry employed compression of these vessels in mania, headache, vertigo, &c. When, however, we consider the irremediable disorganization which is often present in obstinate cases, the difficulty of assigning in many the precise causes of their production, and the temporary character of the remedy, by reason of the quick re-establishment of the circulation by anastomosis, we are surprised that so careful a practitioner as Dr. Burrows should recommend this operation.

"Although the ligature of the common carotid is attended with risk to life in some cases, (perhaps in the proportion of one death in four operations) still experience proves that, where proper precautions have been taken, the operation is not so dangerous as many suppose. Therefore, in violent and hopeless cases of epilepsy and some kindred maladies, which are characterized by extreme cerebral congestion, it appears to me that, other remedies failing, this operation may be fairly resorted to. I am aware of the responsibility of advocating a remedy attended with risk of life; but are not all our best remedies most violent poisons in the hands of the unskilful? But this truth does not forbid their use to the

---

* An interesting case in which both carotids were successfully tied has been recently recorded by Dr. Ellis in the *New York Journal of Medicine, Sept.* 1845. Pettish Hill, æt. 21, was accidentally shot, *Oct.* 21, 1844. The ball passed from just above the spine of the left scapula along the neck into the mouth, making its exit through the upper lip. There was little hæmorrhage, and he was transported twelve miles in a litter. On the seventh day hæmorrhage from the wound of the tongue occurred, but was readily suppressed by compressing the left carotid. On the eighth day, hæmorrhage recurring, the left carotid was tied below the omo-hyoideus. No unpleasant symptoms followed, but on the eleventh day, when a slight pulsation of the temporal artery was felt, hæmorrhage recurred, and again the next day, being temporarily arrested by pressure on the right carotid and on the orifices of the wound. This, however, caused too much pain to be borne, so that, *four days and a half after* the first operation, the *right carotid* was tied. The only bad symptoms which followed were some dyspnœa and cough, which were relieved by a small depletion and then giving small doses of aconite. At the date *June* 18, 1845, the young man enjoyed comfortable health, and was attending to business. No pulsation could then be felt in either temporal artery.—(*For a Case by Dr. Warren, see our present Periscope.*)

more expert. So may this powerful method of influencing the cerebral circulation be justifiable in aggravated cases of the class referred to, and where the precept of Celsus *satius est anceps remedium experiri quàm nullum,* may be fairly put into practice." P. 79.

*On Apoplectic Coma.*—Upon the nature of coma in apoplexy authors afford us no efficient explanation, Abercrombie confessing his ignorance of it. Dr. Burrows believes that too exclusive an attention has been paid to the recognition of visible physical cause of pressure, and suggests that there are several causes capable of suspending the functions of the brain in the same manner as experiment proves their power in destroying those of the cerebro-spinal nerves. These are 1, pressure on the nervous fibres; 2, division of nervous substance; 3, disorganization of nervous matter; 4, interrupted supply, or deficient momentum of blood in the venous substance; 5, narcotics. Numerous cases have been recorded in which death with all the symptoms of apoplexy has occurred, and yet the brain found to be in its normal condition. Various explanations have been proposed by those who maintain the invariableness of the quantity of the blood contained in the cranium: but Dr. Burrows believes that in such cases a congested state of the vessels, produced either by determination of blood to or its detention in the brain, has existed. Of the three cases recorded by Abercrombie two had been freely bled, and no considerable turgescence of the vessels was observed; but in the third, treated only shortly prior to death, the vessels were turgid and the substance of the organ highly vascular. But, supposing this congested state of the brain be relieved by depletion, and no structural lesion be discovered, how do we explain the occurrence of death? This, Dr. Burrows is disposed to refer to the brain becoming saturated during the suspension of consciousness, and the existence of stertorous breathing, with undecarbonized blood, so that the patient actually dies from the effects of imperfect respiration, and not from pressure or lesion of the brain.

The ordinary explanation of the occurrence of death in *sanguineous and serous apoplexy* by the compression of the brain exerted by the effused fluids, is also deemed erroneous by Dr. Burrows. Portal, Abercrombie, and other high authorities admit that the distinction between serous and sanguineous apoplexy is not a real one, that the serum is only secondary upon pre-existing congestion, and that the symptoms bear no relation to the amount effused. The coma cannot then be dependent upon the effused serum, nor is it so when the extravasated fluid is blood; for large and old extravasations have been found with no coma, while this last has prevailed in cases in which slight and recent extravasation only existed. Two cases, which fell under the author's care, and in which transient coma only manifested itself with copious cerebral hæmorrhage, are related. These and numerous similar ones, prove that "extravasation of blood cannot of itself be looked upon as the cause of the coma, although it is so of the paralysis concomitant with the coma, and which remains after the coma has disappeared." The coma, in the majority of cases, is attributable to *attendant and co-existent congestion.*

"When extravasation of blood within the cranium takes place very slowly, as from the rupture of the diseased coats of an artery, independent of determination of blood to the brain, or general congestion of its vessels, it is highly probable that the effusion does not produce the symptoms of apoplectic coma, but gives rise to some modification of hemiplegia, the paralysis being to a greater or less extent, according to the situation and quantity of effusion. As long as extravasation is actually going on, while the blood is pouring forth, it most probably produces pressure on the surrounding cerebral substance, just as the blood escaping from a divided artery in any other part of the body would compress any obstacle with a force equal to the momentum with which the blood was circulating in that vessel. When cerebral hæmorrhage has stopped, I suspect the blood ceases to be a real source of general pressure, although, as it increases the quantity of extra-vascular matter in the cranium, it also offers additional resistance to the entrance of the normal quantity of blood into that cavity; hence healthy vascular distension becomes excessive, and the symptoms of general cerebral pressure are easily developed. Thus, the compression produced by extravasation will depend more on the rapidity and situation of the effusion than on the amount. If the effusion be slow, and not near the base, although the amount be considerable, the effects will be slight in comparison." P. 101.

As the coma may exist without effusion and effusion without coma, in the majority of cases, the coma is therefore due to the pressure induced by vascular congestion. Other causes may induce coma, as for example the circulation of venous blood through the brain; but this is the explanation offered of the coma of apoplexy. That ordinary causes of determination of blood to the brain, such as violent exercise, vivid emotions, and intemperance, do not oftener lead to the production of coma may be attributed to the anatomical provisions for preserving the uniformity of pressure and an easier circulation of blood provided by the tortuosity of the arteries and the arrangement of the sinuses of the dura mater; while the facility with which the extra-vascular serum escapes into the spine is an additional protection against the effects of such sudden congestion.

II. *On the Connection of Apoplexy and Hemiplegia with Diseases of the Heart.*—Dr. Burrows believes the profession is by no means sufficiently aware of the extent of the influence which diseases of the heart exert in inducing functional and structural diseases of the brain. If the explanation of the cause of apoplectic coma be correct, it may be expected that a diseased condition of the heart or its valves, so commonly found coexistent with congestion in other parts of the body, will be frequently present. But none of the standard writers on this disease seem to have noticed such connection prior to Portal, who was much in advance of his German and English cotemporaries. Drs. Kellie and Clutterbuck deny the connection between the diseased condition of the one organ and that of the other, and Dr. Abercrombie seems entirely to have overlooked it. Bright and Andral however admit this connection, but it is by Dr. Hope that it has been placed in the strongest light. He believed, with M. Bouillaud and Bertin, that hypertrophy of the heart predisposes more strongly to apoplexy, than even what is termed the apoplectic constitution.* Richerand agrees in

* At a subsequent page (143) Dr. Burrows has an interesting note upon this so-called constitution. He says—

" It is a popular belief that persons with a peculiar conformation of the body, namely with large heads, red faces, short necks, and capacious chests, are predisthis opinion; and Dr. Copland admits that the lesions co-exist too frequently to be explained by mere coincidence.

Amid such difference of opinion Dr. Burrows has endeavoured to form a statistical account of the frequency of disease of the heart in cases of apoplexy and hemiplegia; but has found this a matter of difficulty, inasmuch as few authors have afforded precise information as to the condition of the heart in this affection, being content with recording the lesions they have met with in the brain. However, from such sources as furnished the necessary data, and from cases observed by himself, he has drawn up the following table of 132 cases.

| | | Diseased Heart. |
|---|---|---|
| Andral .. .. | 25 | 15 |
| Clendinning .. | 28 | 15 |
| Hope .. .. | 39 | 27 |
| Burrows .. .. | 34 | 23 |
| Guillemin.. .. | 6 | 4 |
| | 132 | 84 |

From this, it would seem that three-fifths of a given number of cases of apoplexy or sudden hemiplegia manifest distinct signs of disease of the heart. M. Rochoux, however, states that, of 42 cases examined by him prior to 1818, three only presented hypertrophy; but upon examination of the thirty cases detailed in his work, Dr. Burrows finds that the state of the heart was examined only 14 times, and in four of these there was disease of either the muscular or valvular structures. It is to be remembered too that lesions of the heart were very imperfectly appreciated prior to 1818, and that M. Rochoux restricts the term apoplexy to cases in which actual hæmorrhage is discoverable. In reference to the question of which of the cardiac lesions most frequently gives rise to cerebral affections, Dr. Burrows states that, of the 38 instances of diseased heart observed by Andral and himself in 59 cases of apoplexy and hemiplegia, 19 were examples of hypertrophy with valvular disease, 10 of simple hypertrophy and 8 of valvular disease.

"I have thus endeavoured by facts and arguments, to point out the frequent and intimate relation subsisting between structural changes in the heart and these cerebral affections. This relation appears to me in many cases to be that of cause and effect. I have already quoted the opinions of Portal and some others who have entertained a similar view of the pathology of these cerebral

---

posed to apoplexy: and that persons of spare habit with longer necks are exempt from that disease; so that if a person of this latter description is attacked with apoplexy or hemiplegia, considerable surprise is expressed. My experience causes me to doubt the accuracy of these opinions. The former class of individuals are usually the subjects of considerable hypertrophy of the heart, and hence suffer from habitual determination of blood to the brain, and perhaps hypertrophy of that organ. No wonder that they should suffer from attacks of apoplexy. But I have met with many instances of apoplexy and hemiplegia among the poor where the individuals have been pallid, and attenuated with slight figures; in fact, presenting the very reverse of the so-called apoplectic make; and in such cases, upon making a careful scrutiny of the heart and lungs, I have discovered signs of valvular disease in the heart, or perhaps of extensive emphysema of the lungs; and these diseases probably combined with changes in the arterial coats."

affections; but I am not aware that any other author has presented so connected and extended a review of the arguments and facts, which ought to have the effect of establishing a pathological doctrine of great importance in its application to the treatment of these disorders of the brain.

"Although I have thus maintained the paramount influence of the heart, both in its healthy and diseased states, upon the circulation and functions of the brain, still I am fully sensible that lesions of other organs, especially of the lungs, kidneys, and liver, have a similar, though less frequent and direct, influence in disordering the cerebral circulation, and producing apoplexy, hemiplegia, and epilepsy.

"In opposition to the opinions entertained by many respectable authorities, that the quantity of blood within the cranium is at all times nearly the same, and that the heart does not influence the cerebral circulation, my own observations, supported by facts already detailed, convince me that in many, perhaps the majority, of cases of apoplexy and hemiplegia, the primary disease is not situated within the cranium. I would go further, and affirm, that in many cerebral affections apparently depending on effusions of serum or blood, there is no farther primary disease of the brain than there is of the cellular tissue in anasarca, or of the peritoneum in ascites, or of the skin in purpura, or of the stomach in hæmatemesis. There is, indeed, a palpable morbid condition of these several tissues and organs where the effusion or ecchymosis takes place: but it is generally dependent upon a morbid state of some other viscus, which greatly interferes with the circulation in the parts where the effusions are detected. An hypertrophied left ventricle, or valvular obstruction in the heart, will lead to lesions within the cranium similar to those observed in the stomach and peritoneum, when there is obstruction to the circulation through the portal veins in the liver.

"If the pathology of the brain in apoplexy and hemiplegia be analogous to that of other organs which suffer from effusions of serum and blood, how much must this knowledge improve the routine treatment of apoplexy, which has so extensively prevailed. Does not this view of its pathology render more intelligible those different varieties of the disease, which are described by ancient writers, although they could not account for the differences?" P. 125.

*Period of Life most prone to Apoplexy and Hemiplegia.*—Dr. Burrows observes, that the statements of authors as to the liabilities of different ages are mostly erroneous in not being compared with the numbers living at such periods of life. He institutes an analytical comparison of this kind in 215 well-marked cases; whence it appears that the number of apoplectic cases increases in each successive decennial period from 20 to 70 years of age, while the numbers living gradually diminish. From the researches of Dr. Clendinning it also appears that the proportionate weight of the heart increases with advancing life—so that hypertrophy of that organ is a change which is concomitant to the period of life when apoplexy is most prevalent. Dr. Clendinning has moreover shewn that, while the average weight of the adult brain, when the heart was healthy, was 50·5 ounces, in diseases of the heart it was 52·5 ozs.—a condition he regards as the effect of the cardiac disease. Even a moderate, temporary, congestion of a brain already abnormally large may easily induce an apoplexy; and in persons of advanced age, the cerebral arteries being diseased in structure, may easily give way to even moderate congestion.

1. *Treatment of Apoplexy and Hemiplegia.*—During the *attack* more attention to *raising the head* than is usually bestowed is desirable, for its power of depleting the cerebral vessels is, as proved by the author's experiments, very considerable. In deciding upon the propriety and extent of *bloodletting*, Dr. Burrows lays the greatest stress upon the importance of the indications derivable from *cardiac auscultation*. Without this, the condition of the *pulse* will be found in many cases to be very perplexing. If no disease of the heart can be discovered, or if this consist in simple hypertrophy, depletion may be carried as far as the cerebral symptoms demand.

"But, suppose the examination of the heart discloses the existence of valvular disease to the extent of obstructing the circulation through its cavities, here the pulse will be a most deceptive guide as to the propriety or impropriety of abstracting blood. If the mitral valve be principally implicated, and allow of re-gurgitation from the left ventricle, the small and irregular pulse so commonly observed with that lesion, would probably dissuade from that free abstraction of blood which the cerebral symptoms might require. If, in another case, the aortic valves be found diseased to the extent of not only obstructing the onward current of blood, but also of allowing regurgitation into the ventricles during its diastole, there will probably be associated with this lesion considerable hypertrophy of the left ventricle. Here will be observed a full and vibrating or thrilling pulse, but a pulse of increased action without real power, and hence a deceptive pulse; and one which, if it be regarded without reference to the structural changes of the heart, would invite to a more copious abstraction of blood than was called for by the general symptoms. In each of these last-mentioned cases greater relief to the symptoms will be obtained by a free local abstraction of blood from the vicinity of the heart (either by cupping from beneath the left mamma, or between the left scapula and spine) than by a much larger depletion by venesection." P. 142.

Auscultation would also dissuade us from large depletion, notwithstanding a hard and full pulse, if by it we detected serious valvular disease or ossific deposits. In other cases we may find dilatation of the cavities of the heart and extensive emphysema of the lungs, and be deterred from aggravating by large depletion the condition of the heart which has induced the cerebral congestion, notwithstanding that great congestion and dyspnœa may be present. "I should suggest the employment of cupping-glasses to the nape of the neck, or between the scapulæ, with the internal administration of stimulant diuretics, diffusible stimulants, and the application of rubefacients to the sternum."

2. *The Treatment during the Stage of Cerebral Excitement supervening soon after the seizure.*—The pain in the head, flushing of face, knitting of brow, &c., together with the more active condition of the circulation, which supervene after recovering from the depletion, &c. employed during the attack, are symptomatic of inflammation commencing around the effused clot, and are generally markedly relieved by local depletion, application of cold, purgatives, spare diet, and quietude. When the heat of scalp has subsided, a blister to the occiput relieves the oppressive headache; and if the patient be not very aged or much exhausted, small doses of mercury, short of inducing ptyalism, are useful. Together with the above symptoms, or after they have disappeared, the patient may be subjected to a spasmodic and distressing neuralgic condition of the palsied limbs. The author has tried numerous local remedies for these severe pains, but with little success. When symptoms of remaining cerebral irritation have continued, he has found slight local depletion from the head and evaporating lotions to the limbs the most useful proceeding, and where symptoms of cerebral irritation have been absent, leeches applied to the limb itself have proved serviceable.

3. *Treatment of Paralysis following Apoplexy.*—When patients have suffered only a slight attack, or have been quickly relieved by prompt treatment, they sometimes incautiously are allowed to put themselves too fast forwards (although still suffering from the palsy), as regards getting up, mental occupation, diet, &c. In this way, they endanger the production of a fresh extravasation, before the first one is encysted, and the softened brain has recovered its consistency and excitement or inflammation, and consequent disorganization of the cerebral substance may too be induced. Some cases illustrative of the ill effects of such imprudence and incautiousness are related.

When sufficient time has been allowed for the restoration of the cerebral substance to its normal condition, we may proceed to endeavour, by means of counter-irritants, to excite the suspended functions of the nerves of the palsied limbs. These are sometimes useful, and always sure to employ the attention of the patient. Dr. Burrows thinks little of the utility of electricity, and still less of that of strychnia, which indeed sometimes changes the wearing pains of the limbs into acute suffering. Regular frictions and well-devised exercises of the limbs are useful.

*Functional Disturbance of the Brain, induced by Diseases of the Heart.*—Not only may cardiac disease give rise to the serious and fatal lesions of the brain now adverted to, but to various other symptoms indicative of disturbed cranial circulation. "Recurring attacks of vertigo, headaches, rushing of the blood to the head, epistaxis, somnolency, nervous irritation, and even insanity, may often be traced to the operation of cardiac disease, which has not attracted the notice of the patient or his medical attendant."

Dr. Burrows relates several cases of *epistaxis* dependent upon the disturbed state of the circulation consequent on heart-disease. The congestion of the cerebral vessels so induced may, in one case (or at different periods of the history of the same case), give rise to an epistaxis, and in another to an internal hæmorrhage. "If the foregoing observations be correct, they impart an additional significance to the occurrence of epistaxis, which is often regarded as an isolated and unimportant symptom. This hæmorrhage may, I believe, often be considered as strictly pathognomic of an obstructed circulation through the heart, as hæmoptysis is symptomatic of tuberculated lungs, or intestinal hæmorrhage of an indurated liver." Dr. Latham first called the author's attention to another class of cases manifesting various cerebral symptoms the chief of which is *headache*, and in whom the original cause of the symptoms is hypertrophy of the heart without valvular disease. These persons have usually been spirit-drinkers; they are liable to profuse hæmorrhages, and ultimately become the subjects of general dropsy. Their faces are usually pallid, and their pulse peculiarly hard and incompressible. Corvisart has forcibly alluded to the aggravated forms of disturbance of the mental and other cerebral functions which attend aggravated diseases of the heart, plunging the patient into despair, and sometimes leading him to suicide.

*Affections of the Brain and Spinal Cord dependent upon Acute Diseases of the Heart.*—The observations hitherto made apply only to the chronic structural changes of the heart; but acute diseases of this organ may also give rise to symptoms so indicative of severe affections of the nervous centres, that the primary cardiac affection may be entirely overlooked even by experienced observers. Several of such cases have been published from time to time, but Dr. Burrows is the first who has assembled any number of them (sixteen) together, and endeavoured to connect them by a satisfactory explanation in extension of the principles already laid down. They consist of examples of rheumatic or idiopathic pericarditis or carditis in which the symptoms were those of inflammation of the brain, delirium, myelitis, insanity, coma, chorea, or tetanus. In respect to this last order of morbid manifestations, Dr. Burrows observes that it behoves us, in idiopathic trismus and tetanus, the pathology of which is so obscure and the treatment so unsuccessful, to carefully examine for this source of eccentric irritation. "It is a melancholy reflection, but I fear a just one, that numbers have perished from these supposed diseases of the spinal cord, when in truth the morbid action has been in the heart, although that has not been detected." Dr. Bright has already (Med. Chir. Trans. vol. 22) signalized the frequent occurrence of chorea as a result of rheumatic pericarditis.

From the detail of these cases it is evident that "the most formidable diseases of the brain and spinal cord may arise from irritation of the nerves of the heart, without any structural change in the nervous centres themselves." Some have supposed that they only occur in connection with rheumatism and its consequent pericarditis; but of the 16 cases here detailed, no rheumatic affection existed in seven. In two or three the pericarditis was idiopathic, and in the others came on in the course of various chronic diseases. Dr. Burrows, without denying the possibility of metastasis to the encephalon, states that, in no one of the 11 fatal cases enumerated was any trace of disease of the brain or its membranes discernible; and in four fatal cases recorded by Dr. Watson, an accumulation of serum beneath the membrane was the only morbid symptom discovered. Dr. Bright, remarking that in the cases of chorea induced by pericarditis, inflammatory action was found on the exterior of the pericardium, as well as on the pleura, infers that the phrenic nerve distributed over these parts is the medium of communicating the irritation to the spinal cord. M. Bouillaud likewise has observed that the nervous disturbance in pericarditis especially occurs when this affection is complicated with diaphragmatic pleurisy. Such cases are, however, not necessarily attended with nervous excitement; while Dr. Burrows has observed a few instances of pericarditis unconnected with pleurisy, in which such existed. Dr. Hope refers the peculiar expression of features attendant upon bad cases of pericarditis to the propagation of the irritation through the pneumogastric nerves to the spine, and its reflection hence to the face through the portio dura. Dr. Watson explains the nervous disorders in these cases simply by the disturbed state of the cerebral circulation induced through the embarrassment of the heart's action by the inflammation of its tissues.

"In collecting and collating the foregoing examples of endocarditis and pericarditis, my object has been to draw attention more closely to a class of cases, the real nature of which is so likely to be overlooked; and to enforce the necessity of an early examination of the heart by means of auscultation, in all obscure and intractable affections of the brain and spinal cord. The advantages of such an examination are rendered very conspicuous by a comparison of the relative mortality of the cases where the cardiac disease was detected, and of those where it was not suspected during life. Of the 16 recorded cases, 11 proved fatal, and only 5 recovered. In 4 of the successful cases the diagnosis of cardiac disease was satisfactory, and only suspected in the 5th. In only 2 of the 11 fatal cases was an affection of the heart detected during life, in 1 it was suspected, and in the remaining 8 cases there was no suspicion of acute disease of the heart until it was revealed by examination after death.

"I shall conclude this section with some observations upon the treatment of these cases. It appears that only 5 of the 16 terminated favourably. In 4 the cardiac disease was detected at an early stage, and remedies employed to control the exciting cause of the nervous symptoms. These remedies were the abstraction of blood by venesection, and by cupping from the region of the heart, the application of blisters over the cardiac region, and the free administration of mercury combined with opium, so as to produce mercurial affection of the mouth. In two other cases the disease of the heart was indeed detected during the life of the patient, but nevertheless it ended fatally. It will be instructive to inquire into the cause of the want of success in the treatment of these two. In the one, although a slight affection of the heart was discovered on the day of the patient's admission, still it was not until two days afterwards, and only 24 hours before the boy's death, that the physical signs indicated the existence of pericarditis, and it was only from that period that the active remedies above described were employed to subdue the cardiac inflammation; but they proved ineffectual. Upon the other occasion the pericarditis was only discovered the day before the patient's death, when his system was already exhausted by general dropsy of some weeks' duration, and by five days' continuance of the peculiar urgent nervous symptoms which sometimes indicate the presence of active cardiac inflammation. The want of success in the treatment of these two cases is sufficiently accounted for by the late period at which the cardiac disease was detected." P. 214.

The very complete analysis we have presented of the contents of this work supersedes any lengthened statement of the value we attach to these. Our readers are in a condition to judge for themselves, and we much mistake if they do not pronounce their verdict in favour of the author. That he has ably expounded an important physiological principle, and exhibited a too neglected pathological condition is plain enough, and the only difference we have with him is one of degree. Like most observers whose attention has been directed to some particular subject, he perhaps extends the application of his doctrines to a larger proportion of cases than a farther investigation will warrant. When too we observe so many instances of heart-disease, the subjects of which manifest no cerebral symptoms whatever, we feel the necessity of caution lest we sometimes mistake a mere coincidence for cause and effect; and we believe that apoplexy is more frequently dependent upon a local diseased condition of the blood-vessels or cerebral pulp than the author seems willing to admit. However, there can be no doubt the connexion of the lesions is in many cases such as he represents it; this forms an important fact always to be borne in mind in the management of such, although the frequency of its occurrence may not be as yet determinable. We can, however, scarcely allow that the attention of well-informed practitioners has not of late been considerably directed to the production of head symptoms by heart-disease, as this has now been an established doctrine for some time past in our medical schools.

